# NIR Laser Photobiomodulation Induces Neuroprotection in an In Vitro Model of Cerebral Hypoxia/Ischemia

**DOI:** 10.1007/s12035-021-02496-6

**Published:** 2021-07-28

**Authors:** Elisabetta Gerace, Francesca Cialdai, Elettra Sereni, Daniele Lana, Daniele Nosi, Maria Grazia Giovannini, Monica Monici, Guido Mannaioni

**Affiliations:** 1 Department of Neuroscience, Psychology, Drug Research and Child Health (NeuroFarBa), Section of Pharmacology and Toxicology, University of Florence, Viale G. Pieraccini 6, 50139 Florence, Italy; 2 ASAcampus Joint Laboratory, ASA Res. Div. - Department of Experimental and Clinical Biomedical Sciences “Mario Serio”, University of Florence, Florence, Italy; 3 Department of Health Sciences, Section of Clinical Pharmacology and Oncology, University of Florence, Florence, Italy; 4 Department of Experimental and Clinical Medicine, University of Florence, Florence, Italy

**Keywords:** NIR laser, Photobiomodulation, Organotypic hippocampal slices, Oxygen and glucose deprivation, Neurodegeneration, Glia activation

## Abstract

Brain photobiomodulation (PBM) is an innovative treatment for a variety of neurological conditions, including cerebral ischemia. However, the capability of PBM for ischemic stroke needs to be further explored and its mechanisms of action remain currently unclear. The aim of the present research was to identify a treatment protocol capable of inducing neuroprotection and to investigate the molecular mechanisms activated by a dual-wavelength near infrared (NIR) laser source in an organotypic hippocampal slice model of hypoxia/ischemia. Hippocampal slices were exposed to oxygen and glucose deprivation (OGD) for 30 min followed by NIR laser light (fluence 3.71, 7.42, or 14.84 J/cm^2^; wavelengths 808 nm and 905 nm) delivered immediately or 30 min or 60 min after OGD, in order to establish a therapeutic window. Neuronal injury was assessed by propidium iodide fluorescence 24 h later. Our results show that NIR laser irradiation attenuates OGD neurotoxicity once applied immediately or 30 min after OGD. Western blot analysis of proteins involved in neuroinflammation (iNOS, COX-2, NFkB subunit p65, and Bcl-2) and in glutamatergic-mediated synaptic activity (vGluT1, EAAT2, GluN1, and PSD95) showed that the protein modifications induced by OGD were reverted by NIR laser application. Moreover, CA1 confocal microscopy revealed that the profound morphological changes induced by OGD were reverted by NIR laser radiation. In conclusion, NIR laser radiation attenuates OGD neurotoxicity in organotypic hippocampal slices through attenuation of inflammatory mechanisms. These findings shed light on molecular definition of NIR neuroprotective mechanisms, thus underlining the potential benefit of this technique for the treatment of cerebral ischemia.

## Introduction

Cerebral ischemia is the third leading cause of death in Europe and North America and, in 70% of non-fatal cases, is the first cause of severe disability [1]. In the recent decades, the pathophysiology of cerebral ischemia has been extensively studied and many drugs that appeared to be promising in animal models did not show the same efficacy in clinical studies [2]. To date, the only possible therapeutic approach is to restore the cerebral flow as soon as possible. Currently, the tissue plasminogen activator (tPA) is the only drug in use, but it must be administered within 3 h from ischemic attack to be effective and, very often, patients do not arrive on time for treatment. Moreover, tPA shows strict rules for inclusion criteria and could be burdened by serious adverse drug reactions [3]. Therefore, novel therapeutic approaches with a wider therapeutic window are often searched in order to ameliorate cerebral ischemia clinical outcomes.

Brain photobiomodulation (PBM) could be a promising innovative treatment for a variety of neurological conditions and a new modality for the neuronal activity stimulation. The main benefits attributed to brain PBM therapy are related to different biological processes, such as improving neuron energy metabolism, stimulating neurogenesis and synaptogenesis, and inducing neuroprotection via anti-inflammatory, anti-apoptotic, and antioxidant responses [4–7]. All these characteristics led to an increasing interest and suggested PBM as a novel non-invasive treatment for cerebral ischemia. Indeed, the beneficial effect of PBM in different animal models of stroke has been recently reported, by using different wavelengths and experimental protocols and analyzing several cellular and molecular pathways activated by PBM [7–9]. Interestingly, transcranial laser therapy has been recently tested on stroke patients in clinical trials, showing that PBM is a safe procedure, in terms of mortality or occurrence of adverse effects [10]. Nonetheless, the capability of PBM as a novel alternative therapy for ischemic stroke needs to be further explored and its mechanisms of action remain to be fully elucidated.

The aim of the present research was to assess a PBM protocol with a dual-wavelength NIR laser source capable of inducing neuroprotective effects and to investigate the molecular mechanisms underlying the treatment-induced neuroprotection in an in vitro organotypic hippocampal slice model of cerebral ischemia. According to the most important factors that influence the outcome of brain PBM therapy (i.e., wavelength, fluence, irradiance, operation mode, treatment duration, and repetition regimen), the modality of irradiation and the technical characteristics of the laser source could be fundamental. In the present study, we used a commercial device widely used in clinics to treat diseases such as pain control, rehabilitation, and sport medicine, with anti-inflammatory action and positive effects on cell energy metabolism that have been demonstrated and studied [11]. In particular, the device applied in this research works through two synchronized sources (laser diodes): the first one is a pulsed laser diode (emitting at 905 nm wavelength), while the second diode (emitting at 808 nm wavelength) can operate in continuous or frequenced mode. The two laser beams work simultaneously and synchronously and the propagation axes are coincident. In particular, pulsed emission has the advantage to allow the use of higher doses, avoiding side effects (due to thermal processes), and to enable adequate doses to obtain the therapeutic effect also to deeper tissues. Moreover, the simultaneous supply of two emissions allows the stimulation of different chromophores at the same time. It was reported that 808 nm radiation is absorbed by cytochrome c oxidase, while 904 nm radiation increases the activity of mitochondrial respiratory chain complexes, thus contemporarily stimulating the mitochondrial respiratory chain at different sites [12]. Indeed, we hypothesize that dual-wavelength stimulation could have more significant effects compared to the ones previously described in literature, thus inducing a greater neuroprotection.

## Materials and Methods

Male and female Wistar rat pups (weight 16 ± 3 g, RGD Cat No.: 2308816, RRID:RGD_2308816) were obtained from Charles River. Animals were housed at 23 ± 1 °C under a 12-h light–dark cycle (lights on at 07:00) and were fed a standard laboratory diet with ad libitum access to water. The experimental protocols were approved by the Italian Ministry of Health (Aut. 176; 17E9C.N.VAS) and the European Communities Council Directive of 2010/63/EU.

### Materials

Propidium iodide (PI, CAS No: 25535–16-4) was purchased from Sigma (St Louis, MO, USA). Tissue culture reagents were obtained from Gibco-BRL (San Giuliano Milanese, MI, Italy) and Sigma (St Louis, MO, USA).

### Preparation of Rat Organotypic Hippocampal Slice Cultures

Organotypic hippocampal slice cultures were prepared as previously reported [13, 14]. Briefly, hippocampi were removed from the brains of 7- to 9-day-old Wistar rat pups (Harlan, MI, Italy), and transverse slices (420 µm) were prepared using a McIlwain tissue chopper and then transferred onto 30-mm diameter semiporous membrane inserts (Millicell-CM catalog number: #PICM03050; Millipore, Italy), which were placed in six-well tissue culture plates containing 1.2 mL medium per well. The slice culture medium consisted of 50% Eagle’s minimal essential medium (Code #ECB2071L), 25% heat-inactivated horse serum (Code #26,050,088), 25% Hanks’ balanced salt solution (Code #ECB4006L), 5 mg/mL glucose, 2 mmL glutamine (Code #G7513), and 3.75 lg/mL amphotericin B (Code #A2942). Slices were maintained at 37 °C in an incubator in atmosphere of humidified air and 5% CO_2_ for 14 days. Before experiments, all slices were screened for viability by incubating them for 30 min with propidium iodide (PI, 5 μg/mL); slices displaying signs of neurodegeneration were discarded from the study.

### Oxygen and Glucose Deprivation (OGD) Exposure in Rat Organotypic Hippocampal Slices

Cultures were exposed to OGD as previously reported in detail [15, 16]. Briefly, OGD was reproduced by exposing the slices to serum- and glucose-free medium saturated with 95% N_2_ and 5% CO_2_ for 30 min at 37 °C in an airtight anoxic chamber equipped with an oxygen gas controller (BioSpherix, New York, USA). The cultures were then transferred to oxygenated serum-free medium (75% Eagle’s minimal essential medium (Cat No.: #ECB2071L); 25% Hank’s balanced salt solution (Cat No.: #ECB4006L); 2 mM L-glutamine (Cat No.: #G7513); and 3.75 μg/mL amphotericin B (Cat No: #A2942)) containing 5 mg/mL glucose and returned to the incubator under normoxic conditions. Neuronal injury was evaluated 24 h later.

### NIR Laser Treatment in Organotypic Hippocampal Slices

**Fig. 1:**
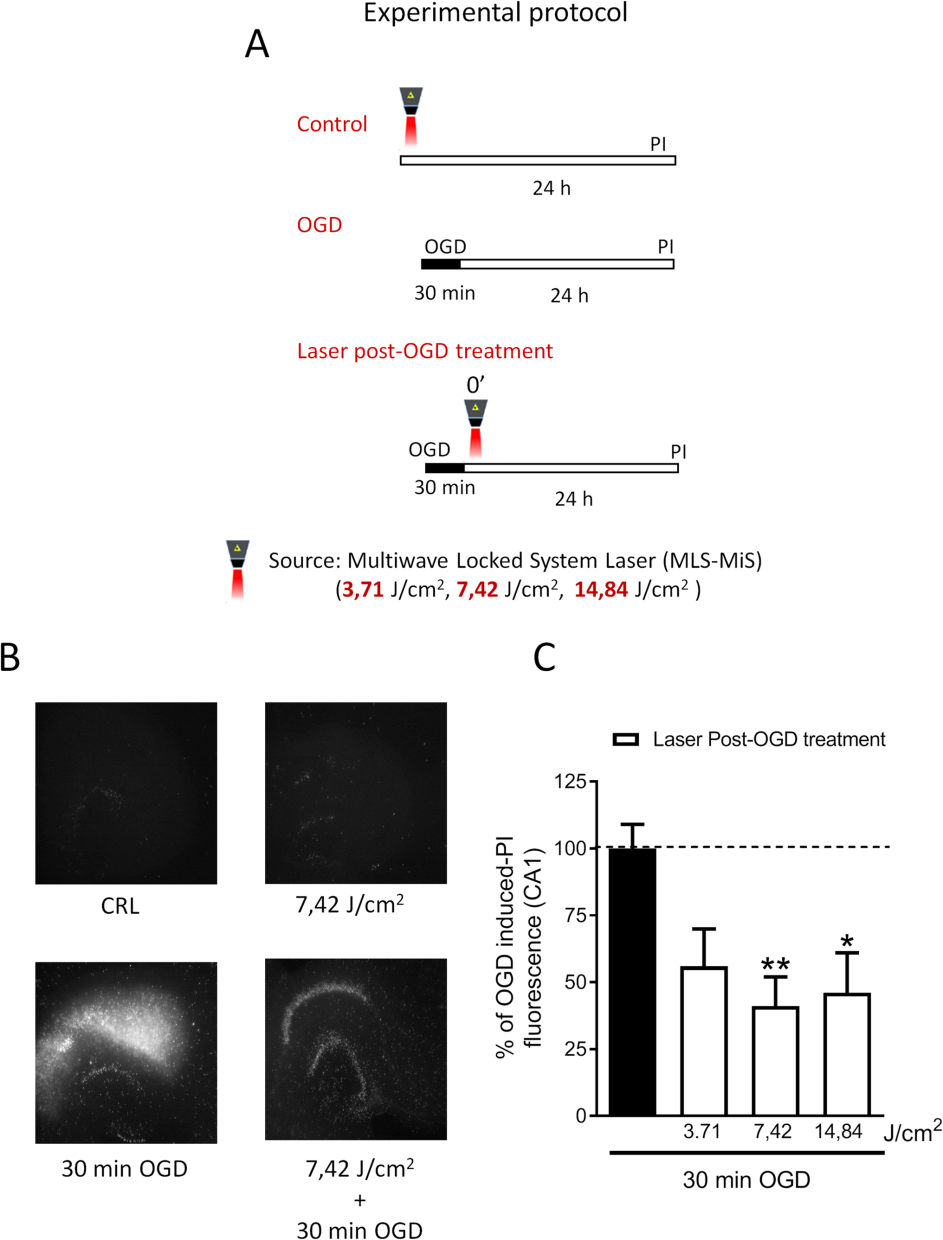
NIR laser treatment induces neuroprotection in rat organotypic hippocampal slices exposed to oxygen and glucose deprivation (OGD). **A** Experimental protocols showing schematic diagrams of NIR laser treatment in organotypic hippocampal slices exposed to 30 min oxygen and glucose deprivation (OGD). **B** Qualitative analysis representing organotypic hippocampal slice in control condition or exposed to 3.71, 7.42, or 14.84 J/cm^2^ alone or immediately after 30 min OGD and 24 h later incubated with PI for fluorescence detection. **C** Quantitative analysis of CA1 PI fluorescence expressed as percentage of OGD toxicity. Data are expressed as percentage of OGD-induced PI fluorescence in CA1 region. Bars represent the mean ± SEM of at least four experiments run in quadruplicate. *P < 0.05, **P < 0.01 vs. OGD (ANOVA + Tukey’s *w* test)

NIR laser treatment in organotypic hippocampal slices was performed with a Multiwave Locked System laser (MLS-MiS, ASA S.r.l., Vicenza, Italy), a class IV NIR laser with two synchronized sources (laser diodes): the first one is a pulsed laser diode emitting at 905 nm wavelength, with peak power from 140 W ± 20% to 1 kW ± 20% and pulse frequency varying in the range 1–2000 Hz; the second laser diode emitting at 808 nm wavelength can operate in continuous (max power 6 W ± 20%) or frequenced (repetition rate 1–2000 Hz, 50% duty cycle) mode. The two laser beams work simultaneously and synchronously and the propagation axes are coincident. The experiments were conducted as described in Fig. 1, panel A. In the first set of experiments, hippocampal slice cultures were exposed once to NIR laser treatment with different exposure time (6, 12, 24 s, respectively) corresponding to fluence (energy delivered per unit area) 3.71, 7.42, or 14.84 J/cm^2^, respectively, alone or delivered immediately after 30 min OGD, in order to evaluate the safety and effectiveness of the different energy doses. After 24 h, neuronal injury was assessed by propidium iodide (PI) fluorescence.

**Fig. 2:**
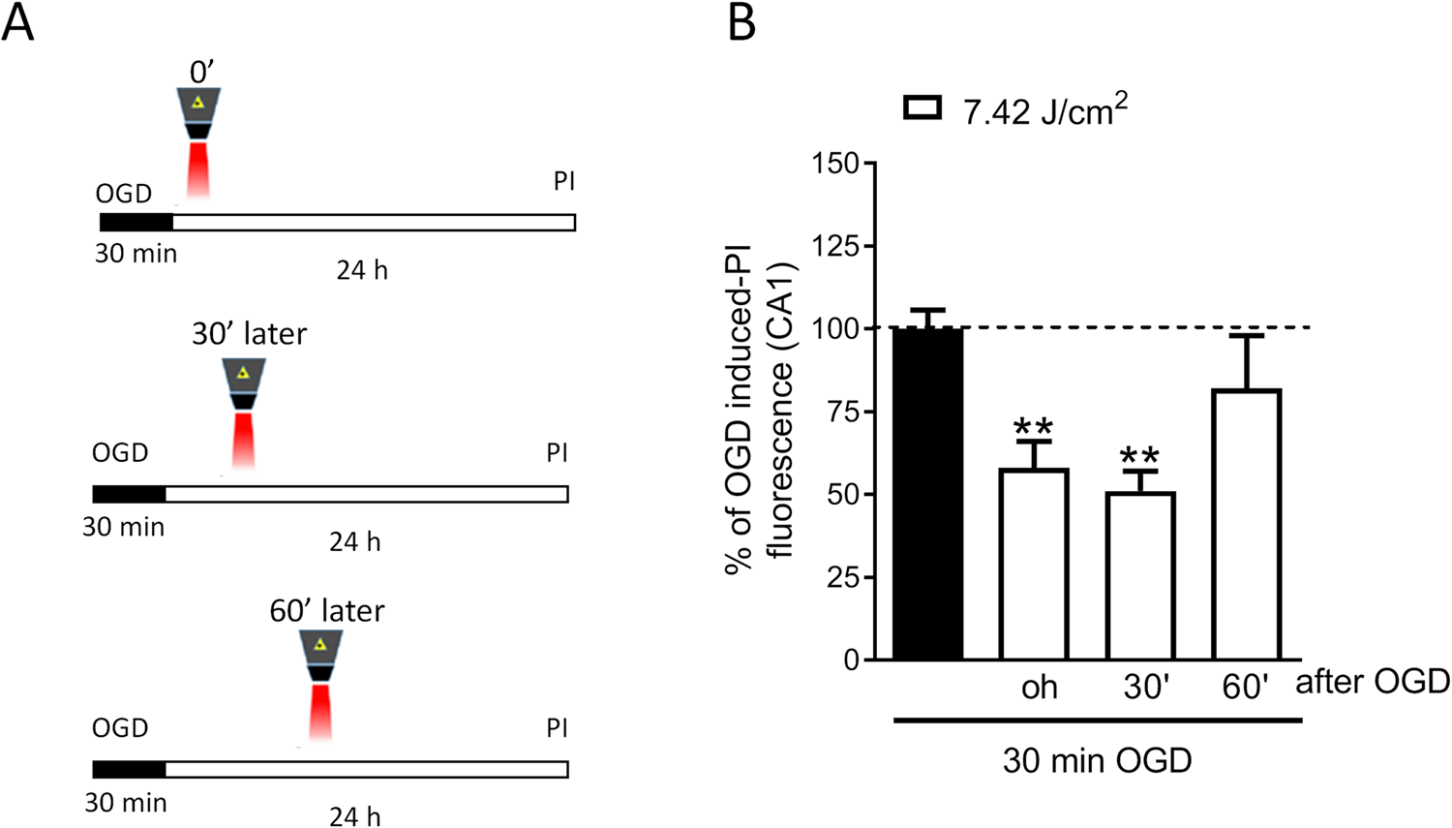
Therapeutic window of NIR laser treatment in rat organotypic hippocampal slices exposed to OGD. **A** Qualitative analysis representing organotypic hippocampal slice in control condition or exposed to 7.42 J/cm^2^ immediately or 30 min or 60 min after 30 min OGD and 24 h later incubated with PI for fluorescence detection. **B** Quantitative analysis of CA1 PI fluorescence expressed as percentage of OGD toxicity. Data are expressed as percentage of OGD-induced PI fluorescence in CA1 region. Bars represent the mean ± SEM of at least four experiments run in quadruplicate. **P < 0.01 vs. OGD (ANOVA + Tukey’s *w* test)

Then, the therapeutic window of NIR laser irradiation was evaluated by exposing the slices to OGD for 30 min followed by NIR laser radiation (7.42 J/cm^2^) delivered immediately or 30 min or 60 min after OGD (Fig. 2). In all laser treatments, the calculated energy density was 620 mW/cm^2^ (1 W = 1 J/s), the pulse frequency 40 Hz, and the intensity 50%, and the 808 source was used in frequenced mode with duty cycle 50% and average power 1.91 W (72% by the 808 nm component and 28% by the 905 nm component), and the size of the laser spot was 3 cm^2^. The hippocampal slices have an approximately elliptical shape, with the major axis of about 3 mm and the minor axis of about 1.5 mm. For the laser treatment, each slice was placed in the center of the spot, with the handpiece held, through a suitable support, in a fixed position perpendicular to the sample.

### Assessment of CA1 Pyramidal Cell Injury

PI (5 μg/mL) was added to the medium at the end of the experiments. Thirty minutes later, fluorescence was viewed using an inverted fluorescence microscope (Olympus IX-50; Solent Scientific, Segensworth, UK) equipped with a xenon-arc lamp, a low-power objective (4 ×), and a rhodamine filter. Images were digitized using a video image obtained by a CCD camera (Diagnostic Instruments Inc., Sterling Heights, MI, USA) controlled by software (InCyt Im1TM; Intracellular Imaging Inc., Cincinnati, OH, USA) and subsequently analyzed using the Image-Pro Plus morphometric analysis software (Media Cybernetics, Silver Spring, MD, USA). In order to quantify cell death, the CA1 hippocampal subfield was identified and encompassed in a frame using the drawing function in the image software (ImageJ; NIH, Bethesda, USA) and the optical density of PI fluorescence was detected. There was a linear correlation between CA1 PI fluorescence and the number of injured CA1 pyramidal cells as detected by morphological criteria [17].

### Western Blot Analysis

The experiments were conducted as previously described in [18, 19]. Cultured slices were washed with cold 0.01 M PBS and dissolved in 1% SDS. Total protein levels were quantified using the Pierce (Rockford, IL, USA) BCA (bicinchoninic acid) Protein Assay (Cat No.: #23,225). A total of 40 μg of proteins was resolved by electrophoresis on SDS–polyacrylamide gel and transferred onto nitrocellulose membranes using the transblot TURBO (Bio-Rad, Hercules, CA, USA). Blots were probed overnight at 4 °C with the specific primary antibodies: polyclonal rabbit iNOS (Code #610,332, BD Transduction Laboratories™, USA), COX2 (Code #15,191, Abcam, UK), phospho p65 (Code #3031, Cell Signaling Technology, USA), Bcl2 (Code #3869, Cell Signaling Technology, USA), vGluT1 (Code #135 303, Synaptic System, Germany), EAAT2 (Code #1783, Abcam, UK), monoclonal mouse PSD95 (Code #MAB1598, Millipore, Italy), and polyclonal goat GluN1 (Code # sc-1467, Santa Cruz, UK), all diluted 1:1000. Immunodetection was performed with secondary antibodies conjugated to horseradish peroxidase. The reactive bands were detected using chemiluminescence ECL reagent (Amersham Biosciences; Cat No: RPN2106). Quantitative analysis was performed using the QuantityOne analysis software (Bio-Rad, Hercules, CA, USA).

### Fluorescence Immunohistochemistry

Immunostaining was performed with the free-floating method [20–22]. Day 1: Organotypic hippocampal slices were placed in a multiwell and washed 3 times for 5 min in PBS-TX, and then blocked for 60 min with BB containing 10% normal goat serum. All antibodies were diluted in BB. Slices were then incubated overnight at 4 °C under slight agitation with a combination of two different primary antibodies: a mouse anti-NeuN antibody (1:400; Code #MAB377, Millipore, Billerica, MA) and a rabbit anti-IBA1 antibody (1:300; Code #016–20,001, WAKO, Osaka, Japan).

Day 2: After three washings in PBS-TX, slices were incubated for 2 h at room temperature in the dark with Alexa Fluor 635 goat anti-rabbit IgG (1:400; Code #A31577, Thermo Fisher Scientific, Waltham, MA, USA) secondary antibody. After three washings in PBS-TX, slices were incubated for 2 h at room temperature in the dark with Alexa Fluor 555 donkey anti mouse IgG (1:400; Code #A31570, Thermo Fisher Scientific) plus Alexa Fluor 635 goat anti-rabbit IgG (1:400; Code #A31577, Thermo Fisher Scientific). After three washings in PBS-TX, astrocytes were immunostained using a mouse anti-GFAP antibody conjugated with the fluorochrome Alexa Fluor 488, for 2 h at room temperature in the dark (1:500 Code #MAB3402X, Millipore). After three washings in PBS-TX, slices were mounted onto gelatin-coated slides using Vectashield mounting medium with DAPI (Code #H-1200, Vectashield, Burlingame, CA, USA).

### Microscopy Techniques and Quantitative Analysis

Confocal microscopy acquisitions were performed in the region of interest (ROI) corresponding to CA1 dorsal hippocampus to acquire immunofluorescence signals of NeuN, GFAP, and IBA1.

Slices were observed under a LEICA TCS SP5 confocal laser scanning microscope (Leica Microsystems CMS GmbH) equipped with a 20 × objective. The parameters of acquisition were maintained constant: frame dimension 1024 × 1024 pixels, frequency of acquisition 200 Hz, z step of 1.2 μm. Quantitative analysis was made with ImageJ software (National Institute of Health, http://rsb.info.nih.gov/ij).

### Evaluation of High Density Nucleus (HDN) Neuron Density

HDN neurons are pyknotic neurons with nuclei that have a highly condensed NeuN-positive nucleus and very faint NeuN-positive cytoplasmic labeling [23]. We counted HDN neurons on 5 different confocal planes equally spaced (4.8 µm) in the depth of the slices. The ROI area was calculated in mm^2^ with ImageJ software and the HDN neuron density was expressed as HDN neurons/mm^2^.

### Evaluation of Astrocyte Density

We counted astrocytes on an internal z-projection of 10 consecutive confocal planes (total thickness 12 µm). The ROI area was calculated in mm^2^ with ImageJ software and the astrocyte density was expressed as astrocytes/mm^2^.

### Evaluation of Reactive Microglia Percentage

We counted reactive and total microglia cells in ROI in accordance with [24] on an internal z-projection of 10 consecutive confocal planes (total thickness 12 µm). Reactive microglia were expressed as percentage of total microglia.

### Statistical Analysis

Data are presented as means ± SEM of *n* experiments from independent cell preparations. Statistical significance of differences between PI fluorescence intensities, immunostaining, or Western blot optical densities was evaluated by performing one-way ANOVA followed by Tukey’s *w* test for multiple comparisons. All statistical calculations were performed using GRAPH-PAD PRISM v. 5 for Windows (GraphPad Software, San Diego, CA, USA). A probability value (*P*) of < 0.05 was considered significant.

## Results

### NIR Laser Treatment Attenuates Neurotoxicity Induced by OGD

As previously reported, organotypic hippocampal slices exposed to 30 min OGD had a selective injury in CA1 pyramidal neurons 24 h later [15, 16]. Figure 1 shows that NIR laser treatment attenuated OGD neurotoxicity. The most effective dose (7.42 J/cm^2^) was able to induce a significant decrease once applied 30 min after OGD, suggesting a therapeutic time window (Fig. 2). Therefore, the 7.42 J/cm^2^ fluence value was chosen for the following experiments. Interestingly, NIR laser light application on control slices did not provoke toxicity by itself at any intensity tested, neither when it was used for 3 consecutive days (once a day) (data not shown).

### Effect of Laser Treatment on the Expression of Neuroinflammatory and Synaptic Proteins

**Fig. 3:**
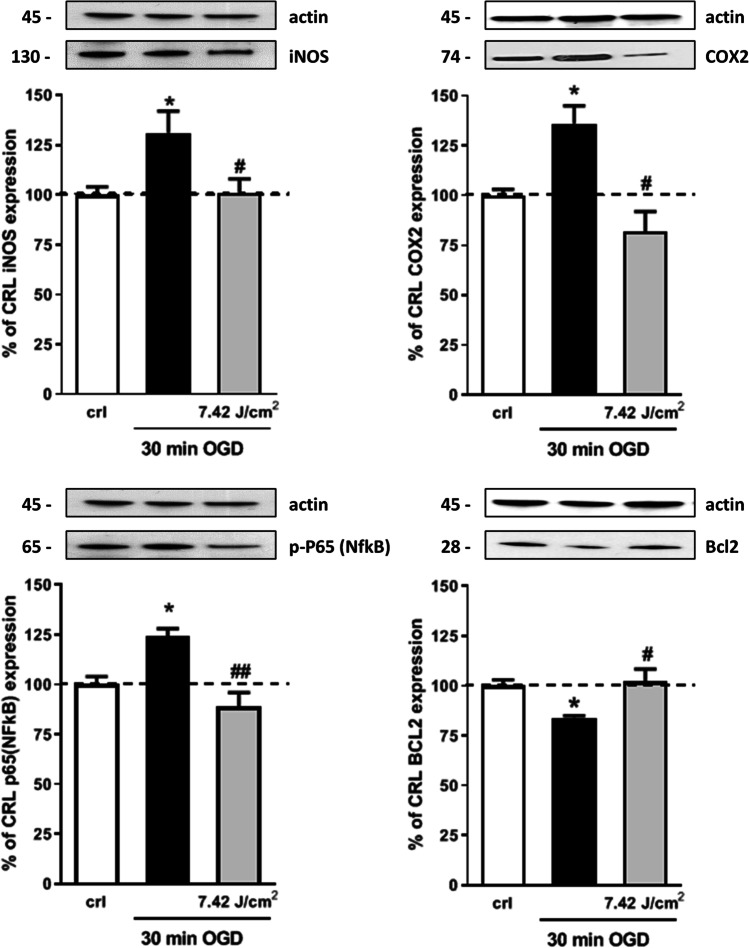
Effects of NIR laser treatment after OGD toxicity on proteins involved in neuroinflammation. Experiments were conducted as described in Fig. 1. Hippocampal slices were exposed to 30 min OGD and immediately after to 7.42 J/cm^2^ NIR laser. Twenty-four hours later, the expression of iNOS, COX-2, phospho p65 (NFkB), and Bcl2 was evaluated in total homogenate by Western blot analysis. Data are expressed as percentage of control. Bars represent the mean ± SEM of at least three experiments run in ottuplicate. *P < 0.05, **P < 0.01 vs. CRL, #*P* < 0.05, ##*P* < 0.01 vs. OGD (ANOVA + Tukey’s *w* test)

**Fig. 4:**
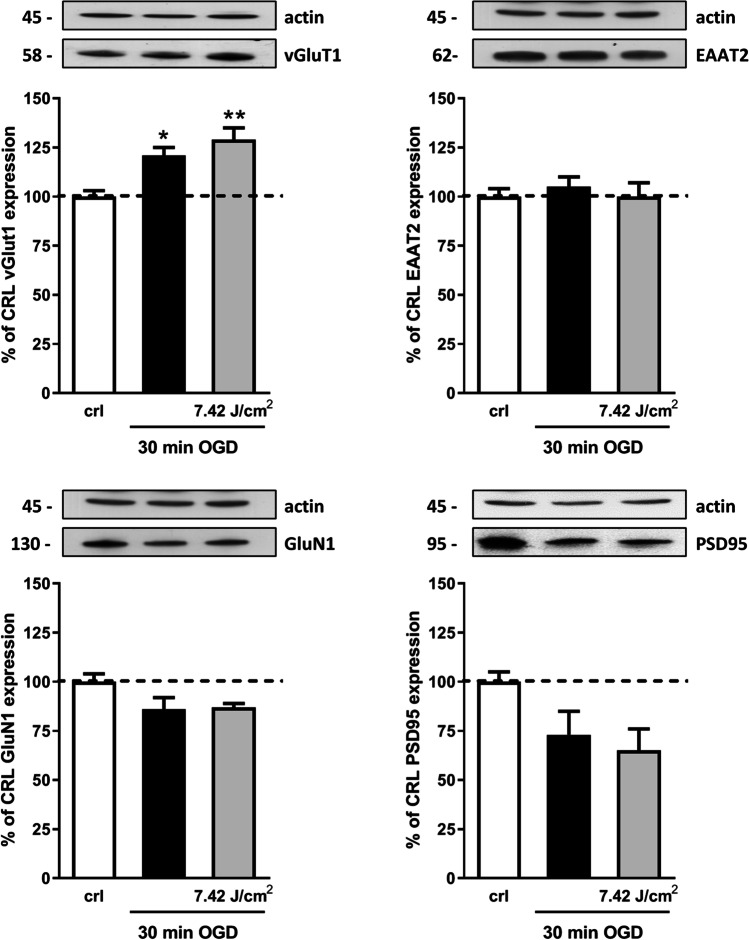
Effects of NIR laser treatment after OGD toxicity on proteins involved in excitatory synaptic activity. Experiments were conducted as described in Fig. 1. Hippocampal slices were exposed to 30 min OGD and immediately after to 7.42 J/cm^2^ NIR laser. Twenty-four hours later, the expression of GluN1, PSD95, vGluT1, and EAAT2 was evaluated in total homogenate by Western blot analysis. Data are expressed as percentage of control. Bars represent the mean ± SEM of at least three experiments run in ottuplicate. *P < 0.05, **P < 0.01 vs. CRL, #*P* < 0.05, ##*P* < 0.01 vs. OGD (ANOVA + Tukey’s *w* test)

To examine in more detail the intracellular neuroprotective mechanisms induced by NIR laser radiation, the pro- and anti-apoptotic proteins involved in neuroinflammation iNOS, COX-2, p65 NFkB subunit phosphorylated form, and Bcl-2 were studied. In particular, post-OGD laser treatment completely abolished iNOS, COX-2, and phospho p65 NFkB subunit over expression, while it reversed the Bcl-2 reduction induced by OGD (Fig. 3). Then, we evaluated whether NIR laser application influenced the expression of proteins involved in excitatory-mediated synaptic activity by analyzing the expression of vesicular glutamate transporter vGluT1, excitatory amino acid transporter 2 (EAAT2), NMDA subunit GluN1, and postsynaptic density protein 95 (PSD95). Western blot analysis revealed that OGD enhanced the expression of vGluT1, but laser treatment did not reverse this effect, suggesting no modification in glutamatergic transmission. Accordingly, no significant changes in the expression of GluN1, PSD95, and EAAT2 were observed after OGD nor after OGD plus laser treatment (Fig. 4).

### Effects of Laser Treatment on Neurons, Astrocytes, and Microglia

**Fig. 5:**
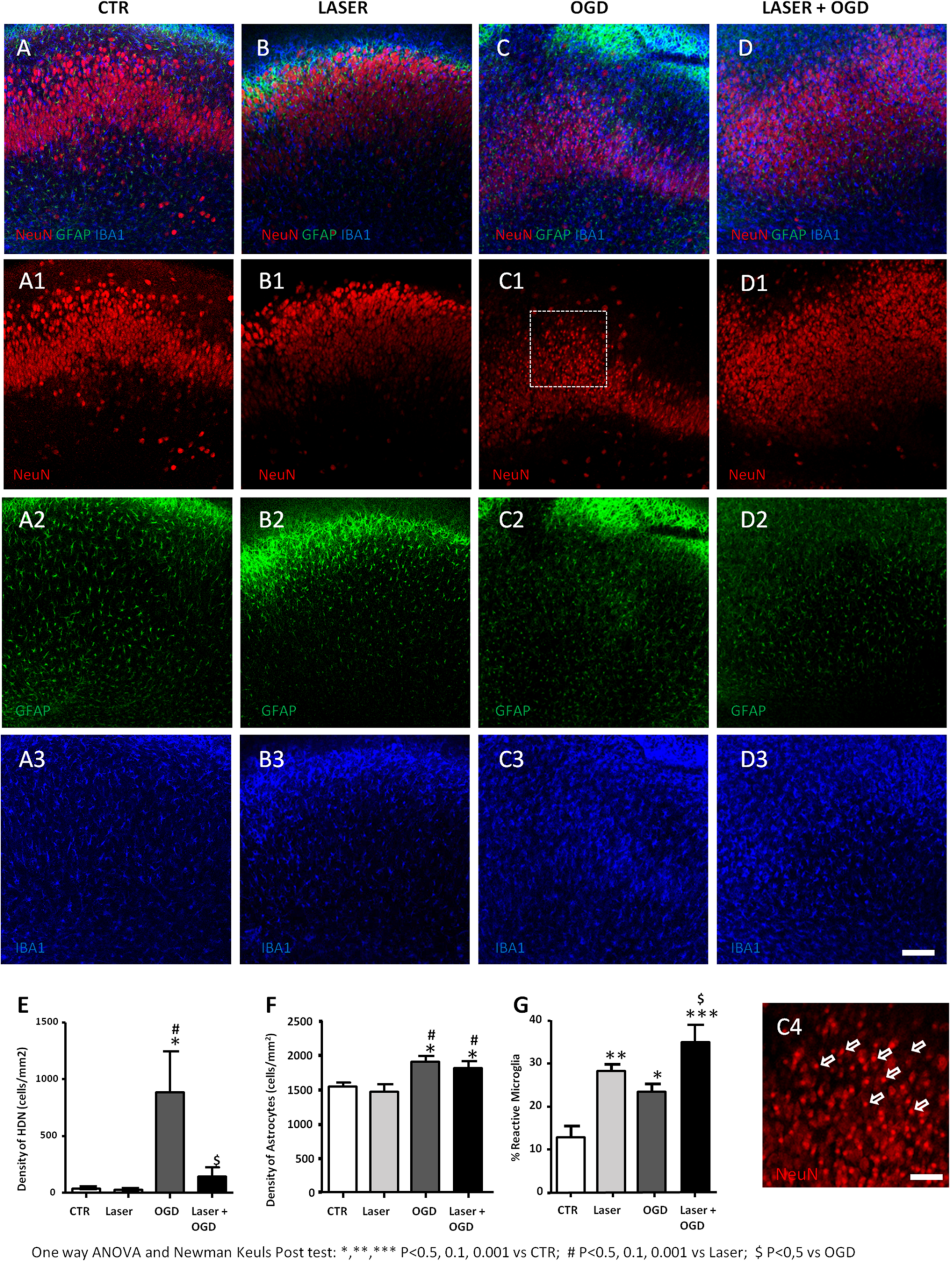
Effects of NIR laser treatment on OGD-induced neurotoxicity and glia activation. **A**–**D3** Representative confocal microscopy images showing CA1 immunostaining of NeuN + neurons (red, **A1**–**D1**), GFAP + astrocytes (green, **A2**–**D2**), and IBA1 + microglia (blue, **A3**–**D3**) in CA1 stratum pyramidale of organotypic hippocampal slices and their merge (**A**–**D**). Control slices (CTR, **A**–**A3**), laser-treated control slices (laser, **B**–**B3**), OGD slices (OGD, **C**–**C3**), and laser-treated OGD slices (laser + OGD, **D**–**D3**). Scale bar: 100 μm. **C4** Magnification of the framed area in C1 showing representative examples of NeuN + pyknotic, and HDN neurons in an OGD slice (open arrows). Scale bar: 40 μm. **E**–**G** Quantitative analyses of NeuN + HDN neurons (**E**), GFAP + astrocytes (**F**), and IBA1 + reactive microglia (**G**) in CA1 of CTR slices (white column, *n* = 6), laser slices (light gray column, *n* = 6), OGD slices (dark gray column, *n* = 7), and laser + OGD slices (black column, *n* = 6) (one-way ANOVA, *P* value = 0.0002; Newman-Keuls post-test: *, **, ***P < 0.05, 0.01, 0.001 vs CTR; #*P* < 0.05 vs laser; $*P* < 0.05 vs OGD)

**Fig. 6:**
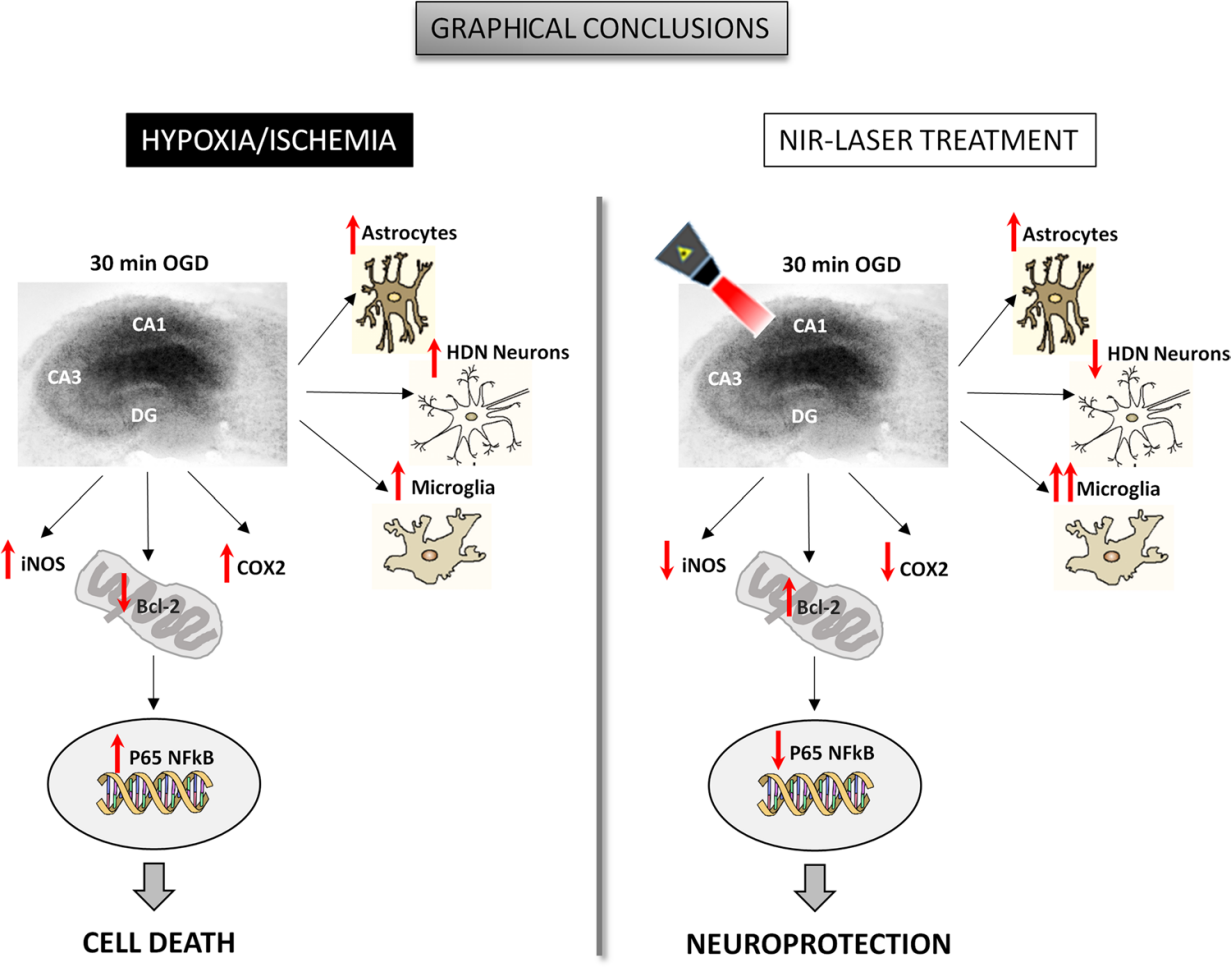
Graphical conclusion: Representative cartoons describing the effects of NIR laser photobiomodulation on organotypic hippocampal slices exposed to hypoxia/ischemia

To characterize the neuroprotective effects of the laser source in CA1 area after OGD, we assessed neuronal viability, astrocyte proliferation, and microglia reactivity using triple immunostaining for NeuN, GFAP, and IBA1 by confocal microscopy. For these experiments, organotypic slices were analyzed 24 h after NIR laser application alone, 30 min OGD alone or OGD plus laser, and in control condition (Fig. 5). Neuronal viability was determined using the quantitative analysis of pyknotic neurons (Fig. 5C4), characterized by a very small, highly condensed nucleus (high density nucleus neurons, HDN), typical of apoptotic cells [25]. Laser treatment alone did not affect the viability of pyramidal cells quantified as HDN neurons (Fig. 5A1–B1 and E), as well as proliferation of astrocytes (Fig. 5A2–B2 and F), as compared to control slices, confirming that PBM by MLS-MiS laser source is safe, not inducing any noxious effect per se. Our results show that 30 min OGD significantly increased HDN neurons in CA1 region (Fig. 5E), confirming the neuronal damage detected by PI staining. Laser radiation was able to significantly reduce the increase of HDN neurons induced by OGD (Fig. 5C1–D1 and E), showing a neuroprotective effect. On the contrary, astrocyte density was not affected by laser application after OGD (Fig. 5A2–D2 and F). Interestingly, quantitative analysis of IBA1 immunostaining showed that the percentage of reactive microglia in CA1 was significantly increased in samples exposed to laser radiation compared to controls (Fig. 5A3–D3 and G). Reactive microglia were further increased when laser irradiation was applied after OGD (Fig. 5C3–D3 and G), indicating that NIR laser emission significantly increased the reactivity of microglia (Fig. 6).

## Discussion

This study aimed to identify a treatment protocol with a dual-wavelength NIR laser source capable of inducing a neuroprotective effect and to investigate the molecular mechanisms underlying the neuroprotection induced by laser treatment in an in vitro organotypic hippocampal slice model of cerebral ischemia. The results obtained show that the application of NIR laser radiation on control slices did not cause toxicity by itself, but it attenuated the neurotoxicity induced by OGD. Most importantly, the efficacy of NIR laser radiation was significant also when it was applied 30 min after OGD, designing a therapeutic time window for the treatment. These data are in agreement with those obtained by other researchers showing that transcranial PBM exerts profound neuroprotective and functional improvement by reducing CA1 injury and by decreasing hippocampal behavioral deficit in experimental models of global ischemia [8, 26]. Moreover, it was previously demonstrated that PBM (continuous wave, 808 nm diode laser, and a dose of 3.0 J) increases the number of surviving hippocampal neurons, reaching approximately 80% of CA1 neuroprotection and preserving both short-term (a week) and long-term (6 months) spatial learning and memory function, after common carotid artery (CCA) occlusion in rats [8]. It is important to notice that the reduction of cerebral ischemic lesions, as well as improved neuronal functionality, was found mainly when PBM therapies were applied within 6 h from ischemic onset [7, 8, 27]. In our study, PBM by NIR MLS-MiS induced a significant neuroprotective effect when applied 30 min after OGD. Considering that our study was conducted in an ex vivo model, where the kinetic of damage is accelerated compared to animal models, the designed therapeutic window is consistent with that reported in the aforementioned in vivo studies, which fixed the time-point to start PBM treatment within 6 h from ischemic onset.

To date, no significant differences were reported when PBM therapy (continuous wave, 830 nm diode laser, 0.28 J/cm^2^ at brain cortex) started 24 h after tMCAO in rats, suggesting that in these experimental conditions PBM does not reduce infarct volume and functional recovery [28]. These results are in line with data obtained by a clinical Phase III (NEST-3) study, concluding that transcranial laser therapy, applied within 24 h (mean time 16 h) after stroke onset, does not have a measurable neuroprotective effect in patients with acute ischemic stroke [29] (NEST-3 clinical study). All together, these results confirm that a defined therapeutic window is of fundamental importance and that further studies are needed to adequately modify the PBM parameters in order to obtain the best protocol for PBM therapy.

Several studies suggested that the molecular mechanisms underlying PBM effects are mainly attributable to the maintenance and preservation of mitochondrial morphology and functions, associated with enhanced neuronal survival and preservation of cognitive and hippocampal functions [8, 30]. For example, Tucker and colleagues demonstrated that transcranial application of PBM (continuous wave, 808 nm diode laser, 3 J/cm^2^) in a rat neonatal model of hypoxic-ischemic injury significantly reduces brain shrinkage and neuronal death. In addition, PBM restores mitochondrial dynamics and decreases both oxidative and activation of mitochondria-dependent neuronal apoptosis [31]. As specified in the technical section, the dual-wavelength laser source (808 nm and 904 nm emissions) used is able to induce a variety of photobiostimulation effects, such as neurogenesis, muscle resistance, and animal motor behavior, described in in vitro and in vivo studies [32–35]. In particular, as previously reported, a pulsed emission (PW) rather than a continuous modality (CW) has the advantage to allow the use of elevated energy pulses avoiding side effects and to enable adequate therapeutic dose to more profound tissues. This was also confirmed by Lapchak and colleagues in a rabbit embolic stroke model where, using varying cortical power density (PW and CW), the authors found that the maximum effect on increased cortical ATP content and reduced embolization was obtained when a PW modality was used [36]. This suggested that the CW energy used in clinical trials [10, 29] could be insufficient to induce the maximal effect on neurons; thus, pulsed treatment regimen may be more effective for tissue survival and overall function [33, 37]. To date, despite the encouraging results obtained in animal models of stroke, the clinical translation of effective transcranial PBM in humans is a big challenge, probably because light energy is strongly attenuated in the passage across the thick human scalp and skull. Therefore, further studies are needed to evaluate different sources and treatment parameters that allow the delivery of a sufficient “dose” of photons at damaged tissue sites, necessary for the development of effective therapeutic devices and treatment protocols.

It is important to underlie that each laser source may activate specific biological cascades depending on several factors, such as wavelength, irradiance, operation mode, treatment duration, and tissue optical properties. In order to better understand the mechanisms of neuroprotection prompted by the NIR laser source applied in the present study, the expression of pro- and anti-apoptotic proteins involved in neuroinflammation (iNOS, COX-2, p65 NFkB subunit phosphorylated form, and Bcl-2) and that of synaptic proteins (vGluT1, GluN1, PSD95, and EAAT2) was analyzed 24 h after OGD or OGD plus NIR laser application. The results obtained showed that post-OGD laser treatment completely abolished iNOS, COX-2, and phospho p65 NFkB subunit overexpression induced by OGD, while it blocked the reduction of Bcl-2 induced by OGD. All these data together indicate that laser treatment caused a reduction of neuroinflammation. Interestingly, no changes were observed after laser treatment on excitatory-mediated synaptic protein expression, suggesting no modification in glutamatergic transmission. Despite the still uncertain mechanisms of action of PBM, many studies have demonstrated that PBM is capable to reduce swelling, increase antioxidants, decrease inflammation, and modulate microglial activation state [26]. The anti-inflammatory effect of the laser source applied in the present study was used also in a model of chronic constriction injury of the sciatic nerve (CCI). The authors demonstrated that the antinociceptive effects, as well as the reduction in mechanical hypersensitivity and spontaneous pain, observed after PBM therapy, are associated with a significant reduction of iNOS expression in the spinal cord [38].

Immunofluorescence analysis of cells located in CA1 showed that NIR laser treatment per se did not affect neuron viability, evaluated using HDN neurons quantitative analysis [23], nor astrocyte or microglia proliferation, as compared to control slices, confirming its safety. NIR laser treatment significantly reduced the OGD-induced increase of HDN neurons, indicating its neuroprotective effect, but had no effect on astrocyte proliferation induced by OGD. Conversely, IBA1 immunostaining showed that the percent of reactive microglia in CA1 was significantly increased after NIR laser application and this increase was further enhanced after OGD. We speculate that NIR laser application could shift the phenotype of microglia polarization from M1 (pro-inflammatory) to M2 (anti-inflammatory) phenotype, leading to anti-inflammatory and neuroprotective effects, in agreement with results obtained by other authors [7].

In conclusion, the present study shed light on the molecular definition of neuroprotective mechanisms due to PBM by a dual-wavelength NIR laser source that may be helpful in designing a future therapeutic application for the treatment of cerebral ischemia. PBM therapy may also be helpful for rehabilitation practice in stroke patients or traumatic brain injury and should contribute to the improvement of care and management of hypoxic/ischemic patients.

## Data Availability

The datasets generated and/or analyzed during the current study are available from the corresponding author on reasonable request.
